# Evobrutinib mitigates neuroinflammation after ischemic stroke by targeting M1 microglial polarization via the TLR4/Myd88/NF-κB pathway

**DOI:** 10.1186/s10020-025-01203-8

**Published:** 2025-04-22

**Authors:** Yixiang Jiang, Ning Wang, Jingyi Liu, Jiayi Li, Lulu Chang, Changxin Yang, Zhengyi Chen, Wei Huang, Jing Wang, Xiujuan Lang, Xijun Liu, Yumei Liu, Bo Sun, Hulun Li

**Affiliations:** Department of Neurobiology, School of Basic Medical Sciences, Harbin Medical University, Harbin, 150081 Heilongjiang China

**Keywords:** Evobrutinib, Cerebral ischemia, Therapy, Microglia polarization, Neuroinflammation

## Abstract

**Background:**

Evobrutinib, a third-generation Bruton's tyrosine kinase (BTK) inhibitor, shows great promise for treating neuroinflammatory diseases due to its small molecular size, ease of absorption, and ability to cross the blood–brain barrier. Although previous studies have confirmed significant BTK expression in microglia, the potential of Evobrutinib to treat ischemic stroke by modulating microglial function and its underlying mechanisms remain to be elucidated.

**Methods:**

Male C57BL/6 mice with cerebral ischemia was established to evaluate the effects of oral Evobrutinib treatment. Assessments included TTC staining, behavioral experiments, and pathological examinations were used to evaluate cerebral ischemic injury. Western Blot, flow cytometry, and qPCR were employed to monitor changes in BTK and pBTK expression in microglia and the impact of Evobrutinib on neuroinflammation following the stroke. In vitro, primary microglia were generated to determine the effects of Evobrutinib on the TLR4/ Myd88/NF-κB pathway and on the polarization of microglial subtypes.

**Results:**

The expression of BTK and pBTK is upregulated in microglia under conditions of cerebral ischemia and oxygen–glucose deprivation (OGD). Evobrutinib treatment not only reduced infarct volume in mice but also ameliorated pathological damage and facilitated neurological function recovery. Flow cytometry revealed that Evobrutinib decreased inflammatory cell infiltration and promoted M2 microglia polarization post-stroke. In vitro studies demonstrated that Evobrutinib downregulated the proportion of pro-inflammatory microglia and curtailed the secretion of inflammatory factors under OGD conditions. Mechanistically, Evobrutinib attenuated the OGD-induced upregulation of TLR4/Myd88/NF-κB expression, an effect that was further enhanced by the addition of the TLR4 pathway inhibitor TAK242.

**Conclusions:**

Evobrutinib inhibits the expression and activation of BTK in microglia, reducing M1 microglia-mediated neuroinflammation and alleviating ischemic injury following stroke. This effect is mechanistically linked to the inhibition of TLR4/Myd88/NF-κB-mediated M1 polarization of microglia.

**Graphical abstract:**

Evobrutinib treatment improves neurological function of mice with cerebral ischemia, and alleviates neuroinflammation by inhibiting M1 microglia polarization through TLR4/Myd88/NF-κB pathway.
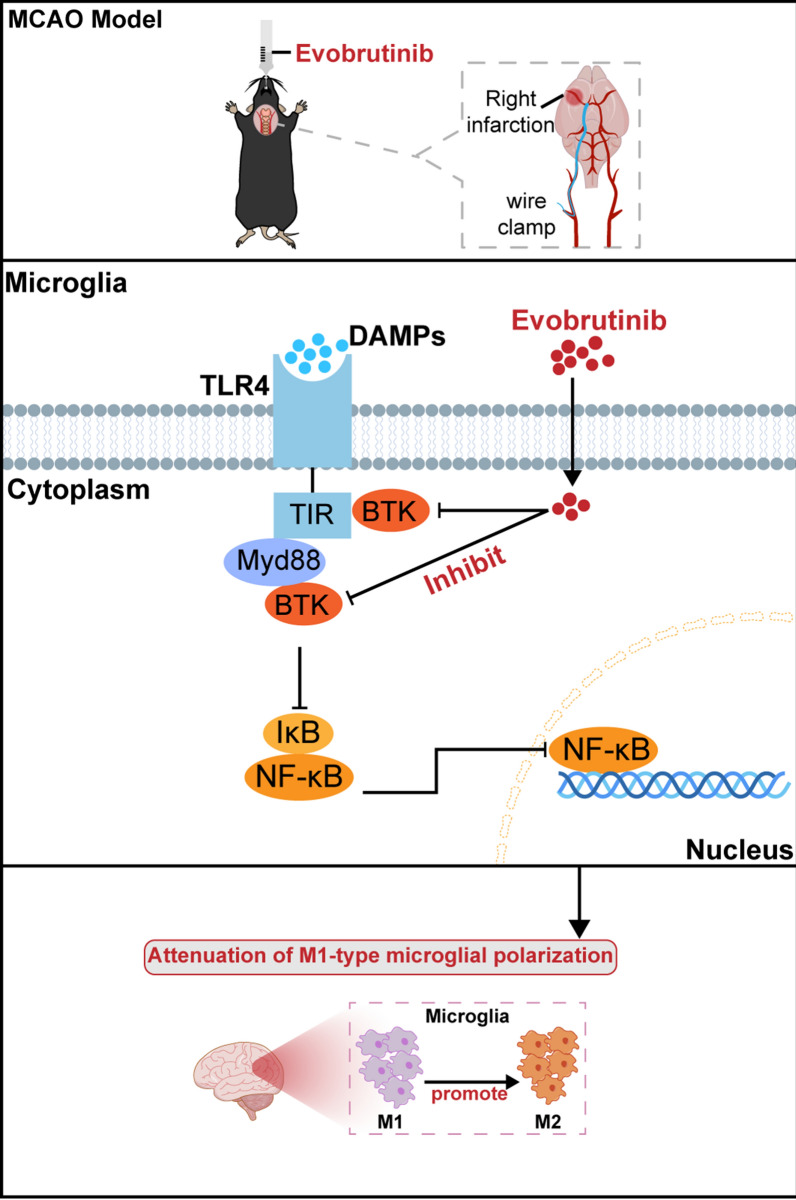

**Supplementary Information:**

The online version contains supplementary material available at 10.1186/s10020-025-01203-8.

## Introduction

Ischemic stroke (IS), characterized by the abrupt occlusion of cerebral arteries leading to localized deficits in glucose availability, hypoxia, and subsequent neurological dysfunction, continues to be a predominant cause of morbidity and mortality globally (Sporns et al. 2022). Clinically, conventional interventions including pharmacological thrombolysis (tissue plasminogen activator, t-PA) and mechanical thrombectomy have moderately reduced the rates of disability and mortality (Tanaka and Reeves 2023). However, the efficacy of vascular recanalization is constrained by a stringent therapeutic window and specific clinical indications, resulting in only approximately 10% of patients deriving benefit (Jovin et al. 2015). Moreover, even among those who undergo successful endovascular therapy, many still experience significant and often irreversible neurological impairments (Misra et al. 2023). Consequently, the identification and development of effective neuroprotective agents to enhance outcomes in ischemic stroke remain a critical and challenging area of research.

Secondary neuroinflammatory responses following IS constitute a critical pathological mechanism that exacerbates brain injury and impedes neurological functional recovery (Sienel et al. 2023). In previous clinical trials, traditional neuroprotective agents (e.g., free radical scavengers and statins), although capable of partially alleviating acute-phase oxidative stress, have demonstrated limited clinical translation due to multiple inherent limitations. These include insufficient blood–brain barrier (BBB) penetration efficiency, narrow therapeutic time windows (< 6 h), and failure to target the neuroinflammatory peak phase (24–72 h post-stroke) (Paul and Candelario-Jalil 2021; Haupt et al. 2023). Consequently, there is an urgent need to develop novel neuroprotective agents with enhanced brain-targeting capabilities and extended anti-inflammatory therapeutic windows to enhance clinical therapeutic outcomes for IS.

Research has demonstrated that the inflammatory cascade initiated by ischemic stroke is activated within minutes following the ischemic event, involving complex interactions among multiple signaling pathways and cellular components (Qin et al. 2022). This includes not only the disruption of the blood–brain barrier (BBB) allowing extensive infiltration of blood-borne inflammatory cells but also the immediate response of the brain’s innate immune cells, the microglia (Candelario-Jalil et al. 2022). Under normal conditions, microglia perform immune surveillance by continuously and rapidly extending and retracting their processes to monitor synapses (Qin et al. 2019). In response to ischemia stroke, microglia swiftly migrate to the lesion's periphery, becoming central to the initiation, progression, and resolution of inflammation (Jia et al. 2022). When triggered by danger-associated molecular patterns released from neurons and other cells, activated microglia alter their gene expression patterns and display two distinct functional phenotypes (Kim et al. 2022). The M1 phenotype secretes pro-inflammatory cytokines (such as interferon-gamma, tumor necrosis factor-alpha, and interleukin-1 beta) and chemokines, which recruit peripheral immune cells and exacerbate the inflammatory response (Li et al. 2023). Moreover, M1 microglia contribute to the degradation of the BBB integrity through the release of reactive oxygen species, nitric oxide, and matrix metalloproteinases, further intensifying ischemic damage (Liu et al. 2020a). Conversely, the M2 phenotype predominantly secretes anti-inflammatory cytokines (IL-4, IL-10, IL-13) and neurotrophins, such as epidermal growth factor and vascular endothelial growth factor, which mitigate neuronal damage and promote angiogenesis and synaptic remodeling (Luo et al. 2022). Thus, the dynamic equilibrium between M1 and M2 microglia is crucial in the modulation of neuroinflammation following a stroke, making it a significant target for anti-inflammatory therapies post-stroke.

Evobrutinib, a third-generation Bruton tyrosine kinase (BTK) inhibitor developed by Merck, demonstrates improved selectivity and safety compared to second-generation BTK inhibitor. Notably, Evobrutinib exhibits higher kinase specificity, minimal off-target effects, and increased safety profiles (Ringheim et al. 2021). As a small molecule drug, Evobrutinib binds to BTK through both covalent and non-covalent interactions, effectively inhibiting BTK activation (Caldwell et al. 2019). Its favorable pharmacokinetic properties, including efficient absorption and the ability to cross the BBB, render Evobrutinib a promising candidate for the treatment of central nervous system (CNS) disorders (Haselmayer et al. 2019; Arnold et al. 2024; Montalban et al. 2022). BTK, a member of the Tec family of protein tyrosine kinases, is integral to the signaling pathway of the B cell antigen receptor, playing a crucial role in the growth and development of B cells (Liu et al. 2023). In addition to its presence in T cells and NK cells, BTK is expressed in mononuclear hematopoietic cells such as macrophages, where it regulates Toll-like receptor (TLR) signaling and chemokine receptor signal transduction processes, influencing the secretion of antibodies and cytokines (Rip et al. 2019). In the CNS, BTK is also expressed in microglia. Research indicates that BTK transcription levels are upregulated in the brains of Alzheimer’s disease mouse models (Keaney et al. 2019), and its expression in microglia is abnormally increased in demyelinating mouse models, correlating closely with the extent of demyelination (Geladaris et al. 2024). Importantly, in vitro studies reveal that macrophages deficient in BTK exhibit impaired M1-type responses and significantly reduced expression of markers such as iNOS (Gabhann et al. 2014). These findings underscore the potential role of BTK in CNS diseases and microglial polarization.

In the present study, we hypothesize that Evobrutinib may inhibit M1 polarization of microglial cells post-ischemia, thereby mitigating neuroinflammation. Compared to conventional anti-inflammatory agents, which suffer from poor BBB penetration and low kinase-targeting specificity leading to off-target effects and systemic toxicity, Evobrutinib may offer a precision intervention strategy based on microglial polarization phenotypes. By combining the dual advantages of high kinase specificity and CNS-targeting capability, this agent demonstrates potential for achieving homeostatic regulation of neuroinflammation following IS, thereby establishing a more effective therapeutic paradigm.

## Methods and materials

### Animals and models

#### (1) Experimental animals

Eight to ten-week-old male and female C57BL/6 mice (20–22 g) were provided by Liaoning Changsheng Biotechnology Co., Ltd. The experimental animals were housed in an SPF-grade animal room, simulating a day–night cycle, with 12 h of light and 12 h of darkness at 22–24 °C, and were given free access to food and water. Female mice were housed with male mice in a 3:1 ratio, and neonatal mice within 48 h of birth were used to extract primary microglia. Male mice were used to establish the middle cerebral artery occlusion (MCAO) animal model. All experiments were approved by the Ethics Committee of Harbin Medical University.

#### (2) MCAO model establishment

The establishment of this model followed the standard method described by Chiang (Chiang et al. 2011), with the procedure detailed as follows: Mice were anesthetized by intraperitoneal injection of 2% sodium pentobarbital. A midline incision was performed on the ventral side of the neck, and the muscle groups were bluntly dissected to sequentially expose the right common carotid artery, external carotid artery, and internal carotid artery. A small incision was made in the external carotid artery to introduce a filament (MSMC21B120PK50, RWD, Shenzhen, China), which was carefully positioned at the middle cerebral artery and left in place. During the procedure, mice’s body temperature was continuously monitored and maintained at approximately 36 °C. Mice exhibiting a Longa score of approximately 3–4 following MCAO surgery were included in the experiment, while those with evidence of subarachnoid hemorrhage or basilar artery rupture were excluded.

#### (3) Pharmacological intervention

Postoperative animals received Evobrutinib (GLPBIO, USA) via gavage for three consecutive days. The dosage was determined based on clinical data, indicating that a daily oral dose of 75 mg was effective in multiple sclerosis patients (Dispenza 2021). Cross-species scaling was conducted using the body surface area normalization model proposed by Nair and Jacob (2016), yielding a final dosage of 10 mg/kg. The administration time remained consistent each day.

### Neurological deficit scores and behavioral tests

The following experiments were conducted in a blinded manner, with the experimenters unaware of the group assignments of the mice.

#### Neurological deficit scores

The neurological function was scored using the Longa five-point scale (Longa et al. 1989), with higher scores indicating more severe behavioral impairment. A score of 0 indicates no neurological deficit; 1 indicates failure to fully extend the contralateral forepaw; 2 indicates circling to the paralyzed side; 3 indicates falling to the paralyzed side; 4 indicates the inability to walk independently and loss of consciousness.

#### Corner turn test

As described elsewhere (Liu et al. 2024), two boards arranged to form a 30° angle were used, and the mouse was placed in the middle. When the mouse advanced to the corner, it would turn either left or right. A complete turn was counted when the mouse's hind legs were fully raised. Each mouse performed the test 10 times, with a 60-s interval between each test. The calculation formula was (number of turns to the healthy side/total number of turns) × 100%.

#### Elevated body swing test

As described elsewhere (Zheng et al. 2024), the mice was lifted by the tail so that its head was approximately 10 cm above the surface. The mouse’s body would swing to the left or right, and a swing angle > 10° was counted. Each mouse performed the test 20 times, with a 1-min interval between each test. The calculation formula was (number of turns to the affected side/total number of turns) × 100%.

### Triphenyl tetrazolium chloride (TTC) staining

Mice were anesthetized and perfused transcardially with PBS. After 3 min, perfusion was stopped, and the brain was removed and placed in a pre-cooled brain mold. Coronal sections of the brain, 1 mm thick, were cut starting from the olfactory bulb. The sections were incubated in 2% TTC solution at 37 °C in the dark for 15–30 min. The sections were gently turned to ensure even staining, photographed, and analyzed for infarct area using Image J software.

### Brain edema

Mice were euthanized, and the brain tissue was quickly removed and weighed wet using an electronic balance. The brain tissue was then dried in an oven at 60–80 °C for 48 h and weighed dry immediately. The percentage of brain water content was calculated as (wet weight − dry weight)/wet weight × 100%.

### Pathological staining

Frozen sections of mouse brain tissue, 10 μm thick, were prepared. HE staining and Nissl staining were performed using hematoxylin–eosin staining solution (G1120, Solarbio) and Nissl staining solution (DK0023, Leagene), respectively, according to the instructions. The sections were dehydrated in graded alcohol and xylene and then mounted with neutral resin. Observations were made using an optical microscope (Nikon, Germany). Immunohistochemistry: Brain tissue sections were blocked with 5% goat serum at room temperature for 2 h. After washing with PBS, the sections were incubated overnight at 4 °C with anti-Neun (ab177487, abcam, 1:1000). The sections were then incubated with HRP-conjugated goat anti-rabbit antibody (ZB-2301, zsbio, 1:1000) at room temperature for 30 min in the dark. After washing with PBS, DAB chromogenic solution (ZLI-9017, zsbio) was added according to the instructions, and the sections were observed under a microscope to monitor the color development. After stopping the staining, the sections were counterstained with hematoxylin for 1 min, dehydrated in graded alcohol and xylene, and mounted with neutral resin. Observations were made using an optical microscope (Nikon, Germany).

### Primary microglia culture

Neonatal C57BL/6 mice (within 48 h of birth) were euthanized. Under sterile conditions, brain tissue was dissected, and the cerebral cortex was isolated, with the meninges and blood vessels stripped as much as possible. The tissue was placed in a 15 ml centrifuge tube containing DMEM/F12 medium (Pricella, Wuhan, China). Using a 5 ml pipette, the tissue was triturated several times and allowed to stand for 3 min before the supernatant was collected and filtered into a new centrifuge tube. This step was repeated until no visible brain tissue remained. The suspension was centrifuged at 1000 rpm at 4 °C for 5 min, the pellet was resuspended in DMEM/F12 medium, and the cells were seeded into culture flasks coated with PDL (Sigma-Aldrich). The cells were cultured in an incubator at 37 °C with 5% CO_2_ (Thermo Fisher, USA) for 15 days. To purify the microglia, the cells were placed on a shaker at 37 °C at 100 r/min for 30 min, 150 r/min for 60 min, and 200 r/min for 30 min. The cell suspension was collected, centrifuged, resuspended in DMEM/F12 medium, and seeded into PDL-coated 6-well plates at approximately 1 × 10^6 cells per well. After 30 min, the medium was replaced to remove non-adherent cells, and the culture continued for 2–3 days until the cell density reached 80–90%, ready for subsequent experiments.

### Establishment of oxygen and glucose deprivation (OGD) microglial model

The culture medium in 6-well plates containing microglial cells was replaced with glucose-free DMEM medium (Pricella, Wuhan, China), supplemented with 1 μM Evobrutinib (GLPBIO, USA) and 500 nM TAK-242 (MCE, USA). The plates were then placed in an anaerobic incubator (PLAS-LABs, USA) at 37 °C with 95% nitrogen and 5% carbon dioxide for 2 h. After OGD treatment, the cells and supernatant were collected for subsequent experiments. The control group was not subjected to any treatment.

### Flow cytometry

Mice were anesthetized, and the spleen was quickly removed, followed by cardiac perfusion with PBS. After 3 min, the brain was removed. The spleen tissue was ground, and red blood cells were lysed to prepare a single-cell suspension. The brain tissue was ground, and 6 ml of 30% percoll solution was added to isolate white blood cells. At room temperature, the suspension was centrifuged at 350×*g* with acceleration and deceleration set to zero for 30 min. The myelin layer and supernatant were removed, and the pellet was washed and resuspended in PBS to prepare a single-cell suspension. Single-cell suspensions from spleen and brain tissues were seeded into 96-well plates at 1 × 10^6 cells/well and incubated at 4 °C for 30 min with PerCP/Cyanine5.5 anti-Mouse CD45 (BD Biosciences, 557235), APC anti-mouse/human CD11b (Biolegend, 101212), PE/Cyanine7 anti-mouse F4/80 (Biolegend, 123113), FITC anti-mouse CD19 (eBioscience, 11–0193-85), PE anti-mouse BTK Phospho (Tyr223) (Biolegend, 601703), and PE anti-mouse Ly-6G (Biolegend, 127607). After OGD, primary microglial cells were collected and incubated at 4 °C for 30 min with APC anti-mouse/human CD11b (Biolegend, 101212), PE/Cyanine7 anti-mouse CD16/32 (Biolegend, 101317), and PerCP/Cyanine5.5 anti-mouse CD206 (Biolegend, 141715). Samples were analyzed using a flow cytometer (BD Bioscience, CA, USA) and analyzed with FlowJo V software.

### Western Blot

Western and IP cell lysis buffer (Beyotime, Shanghai, China) was mixed with PMSF (Beyotime, Shanghai, China) at a ratio of 100:1. The ischemic core region of the brain tissue underwent ultrasonic treatment to prepare a protein suspension. IP cell lysis buffer was added to primary microglial cell culture flasks, and cells were scraped off. Protein concentration was determined using a BCA protein assay kit (Beyotime, Shanghai, China), and protein samples were loaded onto 10% SDS-PAGE gels for separation and transferred to PVDF membranes. The membranes were blocked with 5% non-fat milk at room temperature for 2 h and then incubated overnight at 4 °C with primary antibodies: Rabbit anti-BTK (1:800, ab208937, abcam), Rabbit anti-BTK phospho Y551 (1:800, ab40770, abcam), Mouse anti-TLR4 (1:800, 66350-1, proteintech), Rabbit anti-Myd88 (1:100, A0980, ABclonal), Rabbit anti-NF-κB (1:800, A2547, ABclonal), Mouse anti-GAPDH (1:1000, KTD101, Abbkine), and Mouse anti-β-actin (1:1000, KTD101, Abbkine). The membranes were then incubated with HRP Goat anti-mouse (1:1000, ZB2305, zsbio) and HRP Goat anti-rabbit (1:1000, ZB2301, zsbio) at room temperature for 2 h. ECL chemiluminescence detection was performed using a chemiluminescent imaging system, and protein bands were analyzed using Image J software, with GAPDH and β-actin as internal controls.

### Quantitative PCR

RNA was extracted from the ischemic brain tissue of the mice using the Trizol method (Takara, Japan). Reverse Transcriptase M-MLV (Takara, Japan) was added to the tubes to reverse transcribe RNA into cDNA. The cDNA was diluted with DNase/RNase-free distilled water (Thermo Fisher, USA), and Hieff qPCR SYBR Green Master Mix (high rox plus) (Yeasen, Shanghai, China) and primers were added for real-time quantitative PCR. The ${2}^{{ - \Delta \Delta {\text{C}}_{{\text{t}}} }}$ method was used to calculate the expression of the endogenous control β-actin mRNA. The primer sequences were as follows:

TNFα-F: CAGGCGGTGCCTATGTCTC

TNFα-R: CGATCACCCCGAAGTTCAGTAG

IL1β-F: TTCAGGCAGGCAGTATCACTC

IL1β-R: GAAGGTCCACGGGAAAGACAC

IL6-F: CTGCAAGAGACTTCCATCCAG

IL6-R: AGTGGTATAGACAGGTCTGTTGG

TGFβ-F: CGTGGAAATCAACGCTCCAC

TGFβ-R: GAAGTTGGCATGGTAGCCCT

IL4-F: CCATATCCACGGATGCGACA

IL4-R: CGTTGCTGTGAGGACGTTTG

IL10-F: GGTGAGAAGCTGAAGACCCTC

IL10-R: GCCTTGTAGACACCTTGGTCTT

CX3 CR1-F: GAGTATGACGATTCTGCTGAGG

CX3 CR1-R: CAGACCGAACGTGAAGACGAG

CXCL10-F: CCAAGTGCTGCCGTCATTTTC

CXCL10-R: GGCTCGCAGGGATGATTTCAA

CCL2-F: TGACCCCAAGAAGGAATGGG

CCL2-R: ACCTTAGGGCAGATGCAGTT

CD86-F: CTGGACTCTACGACTTCACAATG

CD86-R: AGTTGGCGATCACTGACAGTT

iNOS-F: GTTCTCAGCCCAACAATACAAGA

iNOS-R: GTGGACGGGTCGATGTCAC

CD206-F: CTCTGTTCAGCTATTGGACGC

CD206-R: TGGCACTCCCAAACATAATTTGA

Arg1-F: CTCCAAGCCAAAGTCCTTAGAG

Arg1-R: AGGAGCTGTCATTAGGGACATC

βactin-F: TGGAATCCTGTGGCATCCATGAAAC

βactin-R: TAAAACGCAGCTCAGTAACAGTCC

### Enzyme-linked immunosorbent assay

After OGD treatment of primary microglial cells, the cell supernatant was collected, and the secretion levels of IL-1β (Invitrogen, 88–7013-22), TNF-α (Invitrogen, 88–7324-88), IL-4 (novus-val603), and TGF-β (novous, VAL611) were detected using ELISA kits.

### Statistical analysis

All experiments were repeated at least three times, and the experimental data were statistically analyzed using GraphPad Prism 9.0 software. Comparisons among three or more groups were performed using one-way ANOVA. P < 0.05 was considered statistically significant.

## Results

### Result 1: Elevated expression of BTK in the brain of mice following cerebral ischemia

**Fig. 1:**
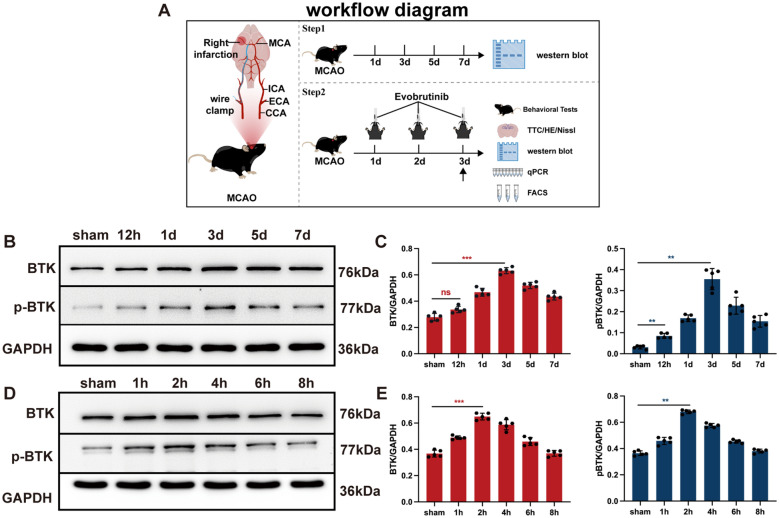
The expression of BTK and pBTK in the brain after cerebral ischemia. **A** Schematic diagram of experimental design. **B**, **C** Expression levels of BTK and pBTK proteins in brain tissues at different time points after ischemia (**B**), along with statistical analysis (**C**). **D**, **E** Expression of BTK and pBTK proteins (**D**) in microglia at different time points in the condition of OGD, along with statistical analysis (**E**). Mean ± SEM, n = 5, one-way ANOVA, ns: no significance; vs sham *P < 0.05, ***P < 0.001

To investigate changes in BTK expression following cerebral ischemia, we utilized C57BL/6 male mice to establish an MCAO model. BTK expression was quantified at various time points post-MCAO (Fig. 1A). Our findings indicate that with prolonged ischemia, both BTK and pBTK levels significantly increased, reaching a peak at day 3 post-MCAO (Fig. 1B, C). Additionally, OGD model was employed to mimic the ischemia-hypoxia microenvironment in vitro. Within this model, BTK and pBTK levels in microglia initially increased OGD duration but subsequently declined, peaking at 2 h (Fig. 1D, E).

### Result 2: Evobrutinib treatment improves brain injury in mice with ischemic stroke

**Fig. 2:**
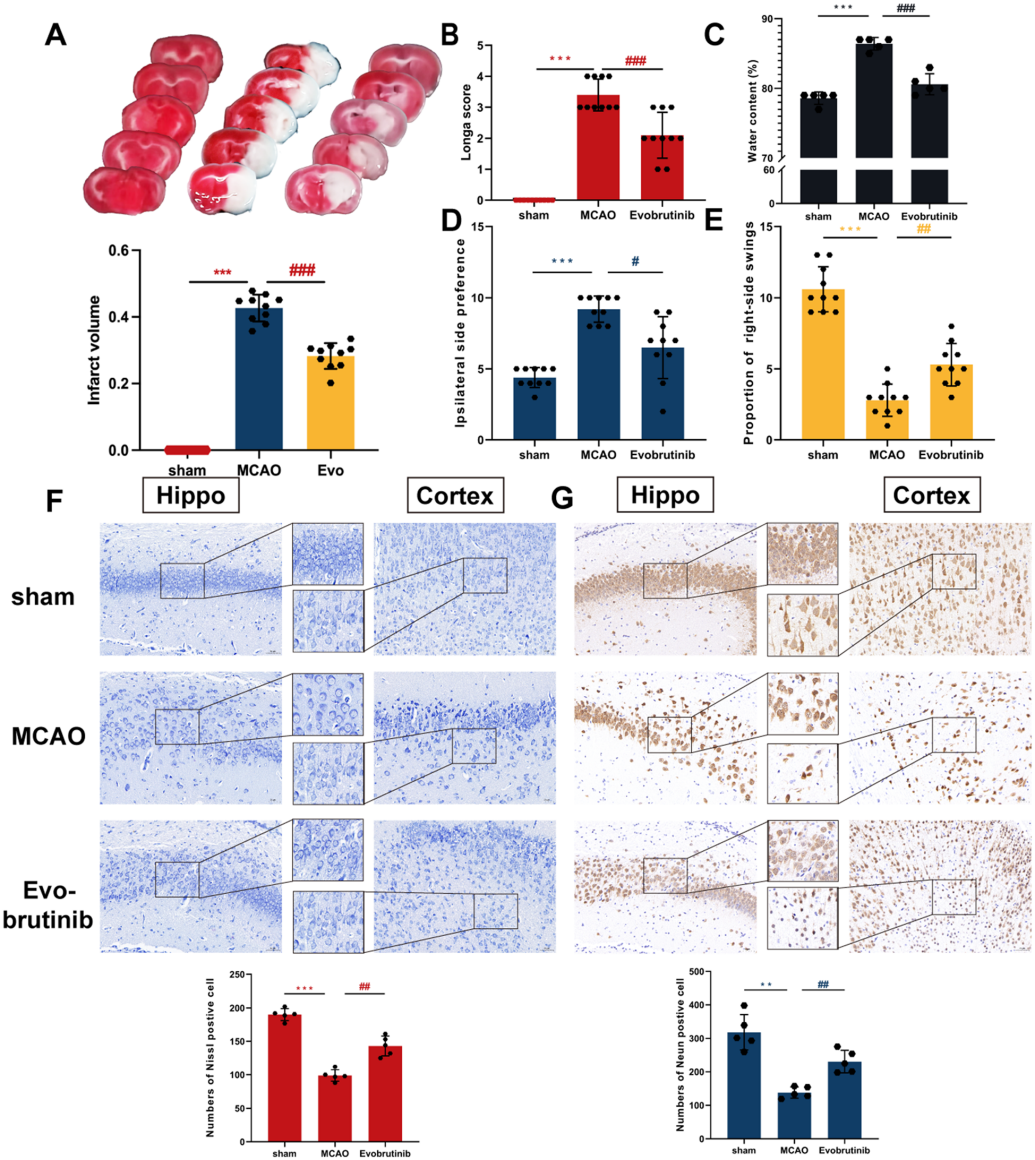
Evobrutinib treatment improves brain injury in mice with ischemic stroke. **A** Brain slices stained with TTC, where normal brain tissue appears red and infarct areas appear white. TTC staining and quantitative data indicate a reduction in infarct volume following Evobrutinib treatment. **B**–**E** Statistical analysis of neurological scoring (**B**), brain edema (**C**), corner turn test (**D**), and elevated body swing test (**E**) demonstrating the impact of Evobrutinib treatment on neurological behaviors in mice with ischemic stroke. **F** Representative images of Nissl staining at ×200 magnification in the hippocampal area (left) and cortical area (right). **G** Representative images of NeuN immunohistochemical staining at ×200 magnification in the hippocampal area (left) and cortical area (right). Mean ± SEM, n = 5 or 10, one-way ANOVA, vs sham *P < 0.05, **P < 0.01, ***P < 0.001; MCAO vs Evobrutinib ^##^*P* < 0.01, ^###^*P* < 0.001

To investigate the efficacy of Evobrutinib in treating ischemic stroke, we administered Evobrutinib orally to MCAO-induced mice. TTC staining demonstrated a substantial reduction in the volume of cerebral ischemia following Evobrutinib treatment (Fig. 2A). Neurological scoring revealed significant improvements in MCAO mice treated with Evobrutinib compared to the sham group, with a notable decrease in neurological deficits (Fig. 2B) and extent of cerebral edema (Fig. 2C). The corner turn test, which assesses asymmetry in sensory and motor functions, showed that normal mice typically turn equally in both directions, whereas MCAO mice exhibit a preference for turning towards the affected side. Treatment with Evobrutinib significantly reduced the number of rightward turns (Fig. 2D). The elevated body swing test, used to evaluate motor functions, indicated that mice with severe neurological deficits turn less frequently; however, treatment with Evobrutinib markedly increased the rate of rightward turns (Fig. 2E). Pathological analysis revealed that Nissl bodies exhibited dense and regularly arranged in normal mice, whereas the MCAO group showed irregularly arrangement and reduced density—both of which were significantly improved following Evobrutinib treatment (Fig. 2F). Furthermore, NeuN immunohistochemical staining highlighted that while neurons in the cortex and hippocampus of control mice were neatly and compactly arranged, those in the MCAO group displayed significant neuronal loss, with irregular and sparse arrangement. This neuronal damage was less pronounced in the Evobrutinib-treated group (Fig. 2G). Collectively, these findings preliminarily suggest that Evobrutinib ameliorates neurobehavioral disorders in MCAO mice and mitigates the pathological damage to ischemic brain tissue, indicating its therapeutic potential for ischemic stroke.

### Result 3: Evobrutinib treatment improves neuroinflammatory responses in mice with cerebral ischemia

**Fig. 3:**
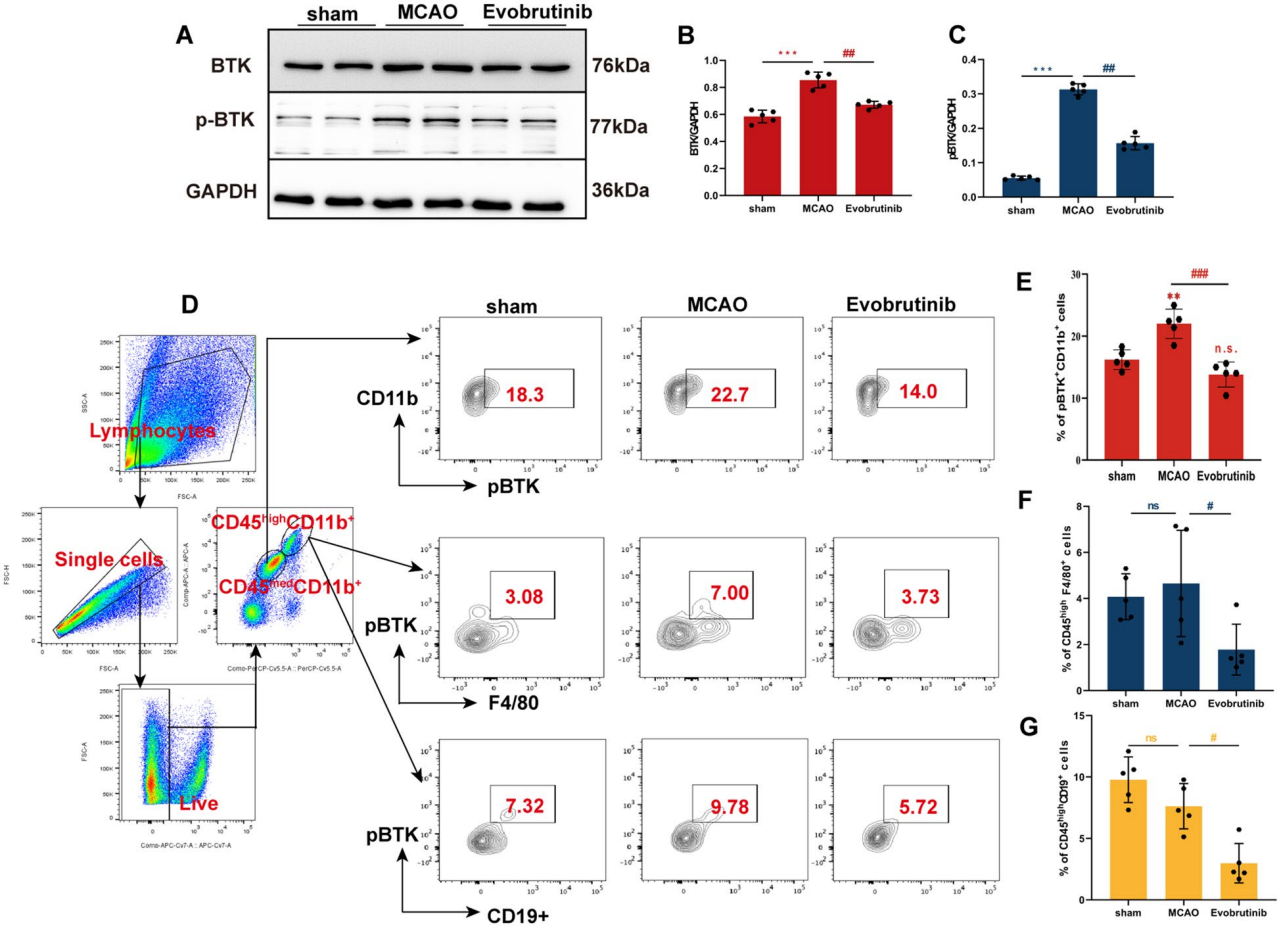
Effect of Evobrutinib treatment on BTK in brain tissues. **A**–**C** Western Blot analysis of BTK expression in the sham, MCAO, and Evobrutinib treatment groups, along with statistical analysis. **D** Flow cytometry detection of pBTK expression levels in microglia, macrophages, and B cells within brain tissues. **E**–**G** Statistical analysis of pBTK expression levels in microglia (**E**), macrophages (**F**), and B cells (**G**) in brain tissues. Mean ± SEM, n = 5, one-way ANOVA, vs sham ns: no significance, *P < 0.05, ***P < 0.001; MCAO vs Evobrutinib ^#^*P* < 0.05, ^##^*P* < 0.01

To elucidate the therapeutic effects and mechanisms of Evobrutinib in ischemic stroke, we initially investigated its impact on target cells. Western Blot analysis revealed that Evobrutinib substantially reduced the expression levels of BTK and phosphorylated BTK (pBTK) in the brain (Fig. 3A–C). Given BTK’s role in various immune cells, we further utilized flow cytometry to assess pBTK expression in microglial cells, macrophages, and B cells within both brain and spleen tissues. The results indicated that Evobrutinib predominantly decreased pBTK expression in microglia (Fig. 3E), infiltrating macrophages (Fig. 3F), and B cells (Fig. 3G) in the brain. Conversely, in the spleen, there was no significant change in pBTK expression levels in macrophages and B cells (Supplementary Figure 1A–C). As innate immune cells of the brain, the modulation of microglial pBTK expression by Evobrutinib may underlie its therapeutic efficacy in treating ischemic stroke.

**Fig. 4:**
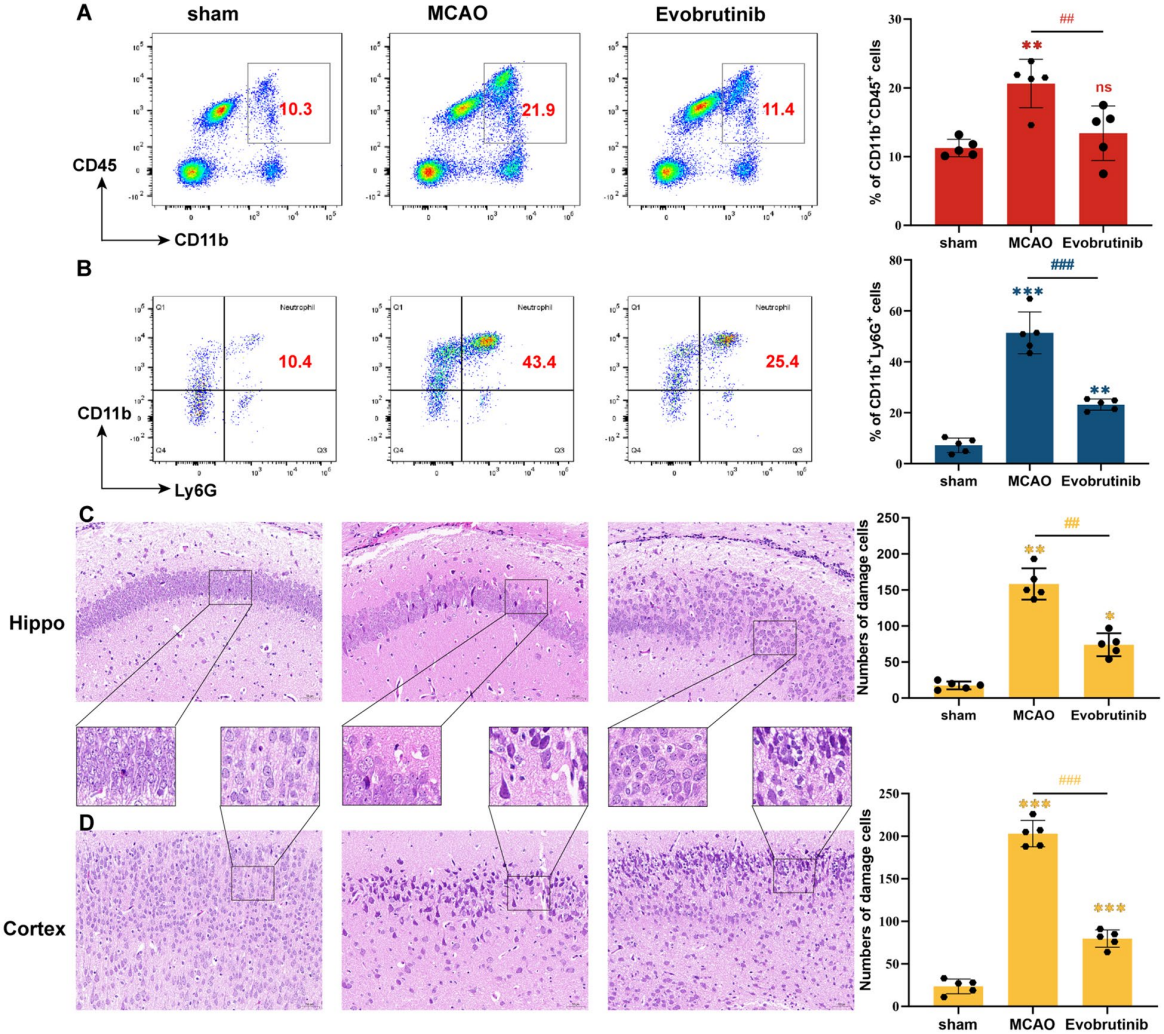
Evobrutinib treatment improves neuroinflammatory response in mice with cerebral ischemia. **A** Flow cytometry analysis of the proportion of CD4^high^CD11b^+^ infiltrating inflammatory cells in the brains of mice from each group. **B** Flow cytometry analysis of the proportion of CD45^high^CD11b^+^ Ly6G^+^ neutrophils in the brains of mice from each group. **C, D**: Representative images of HE staining at ×200 magnification in the hippocampal (C) and cortical (D) areas. Statistical analysis of HE staining demonstrates the impact of Evobrutinib treatment on brain tissues following ischemic stroke injury. Mean ± SEM, n = 5, one-way ANOVA, ns: no significance; vs sham *P < 0.05, **P < 0.01, ***P < 0.001; MCAO vs Evobrutinib ^##^*P* < 0.01, ^###^*P* < 0.001

**Fig. 5:**
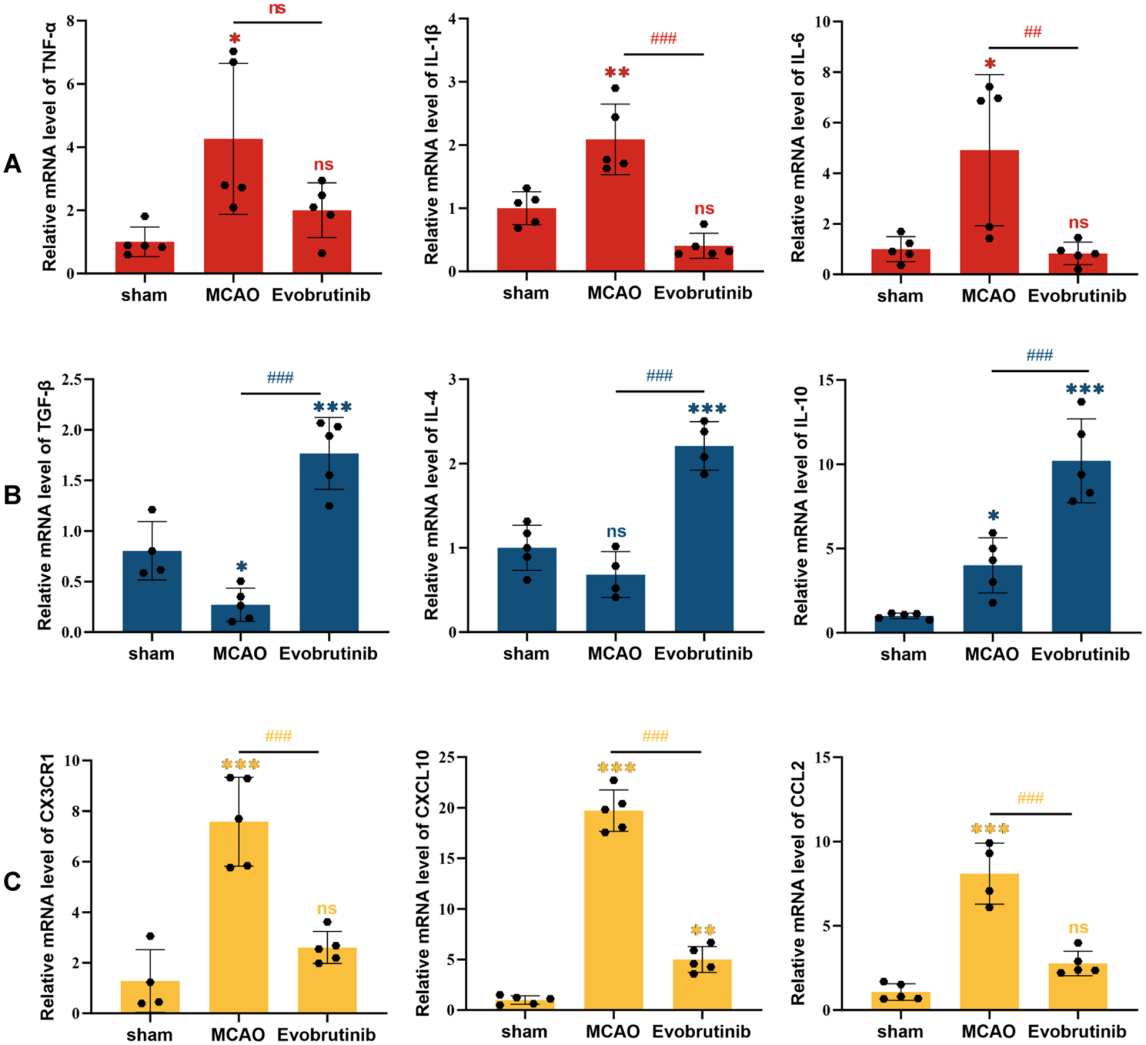
Evobrutinib treatment reduces inflammatory response in MCAO mice. **A** Expression levels of pro-inflammatory cytokines TNF-α, IL-1β, and IL-6 in the brain after Evobrutinib treatment. **B** Expression levels of anti-inflammatory cytokines TGFβ1, IL-4, and IL-10 in the brain after Evobrutinib treatment. **C** Expression levels of inflammatory chemokines CCL2, CXCL10, and the chemokine receptor CX3 CR1 in the brain after Evobrutinib treatment. Mean ± SEM, n = 5, one-way ANOVA, vs sham ns: no significance, *P < 0.05, **P < 0.01, ***P < 0.001; MCAO vs Evobrutinib ns: no significance, ^##^*P* < 0.01, ^###^*P* < 0.001

Subsequently, we investigated the impact of Evobrutinib on neuroinflammation in the brains of ischemic mice. Flow cytometry analysis indicated that Evobrutinib significantly reduced CD45^+^CD11b^+^ peripheral myeloid cells infiltration to levels comparable to the sham group (Fig. 4A). Additionally, the treatment notably decreased the proportion of brain Ly6G^+^ neutrophils (Fig. 4B). H&E staining revealed that in normal mice, brain tissue structure was dense, with cells arranged regularly and nuclei appearing round and prominently blue. In contrast, the MCAO group displayed disorganized cell arrangement, vacuolation in the cortex and hippocampus, and nuclear atrophy, indicative of significant histopathological changes; these abnormalities were substantially mitigated following Evobrutinib treatment (Fig. 4C, D). Furthermore, Evobrutinib significantly reduced the expression of pro-inflammatory cytokines IL-1β and IL-6 (Fig. 5A) and increased the expression of anti-inflammatory cytokines IL-4, IL-10, and TGF-β (Fig. 5B). We also assessed the levels of inflammatory chemokines CCL2, CXCL10, and CX3 CR1 associated with microglial activation. The results demonstrated a significant reduction in the expression of these chemokines post-treatment, with levels not differing significantly from those in the sham group (Fig. 5C). These findings suggest that Evobrutinib inhibits BTK expression in microglia, thereby ameliorating neuroinflammation and potentially offering therapeutic benefits for ischemic stroke.

### Result 4: Evobrutinib treatment reduced the proinflammatory phenotype of microglia

**Fig. 6:**
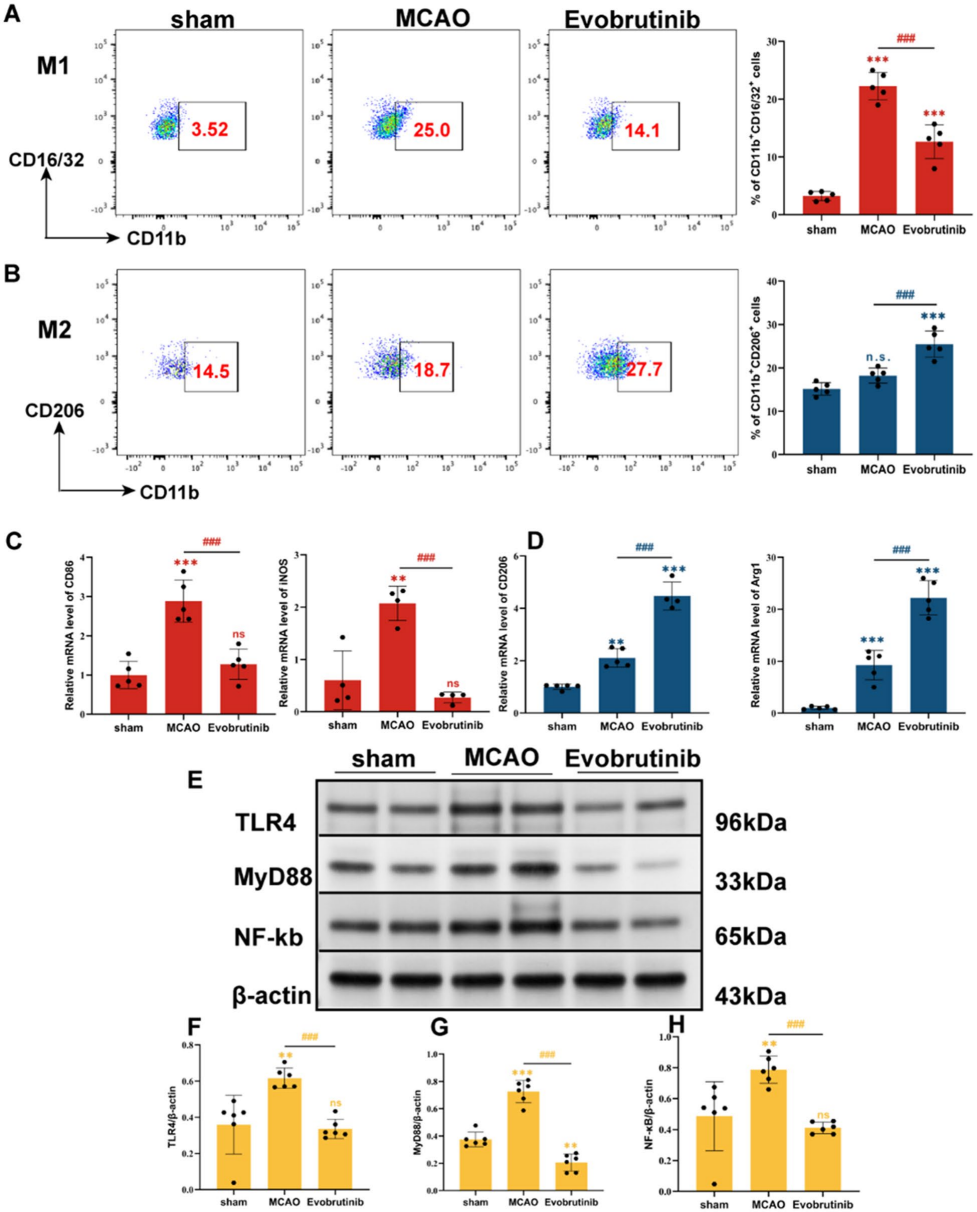
Effect of Evobrutinib treatment on microglial subtypes following ischemic stroke. **A** Flow cytometry analysis of the proportion of M1 microglia in the brain post-stroke. Statistical analysis shows that Evobrutinib treatment reduced the proportion of M1 microglial subtype. **B** Flow cytometry analysis of the proportion of M2-type microglia in the brain post-stroke. Statistical analysis shows that Evobrutinib treatment increased the proportion of M2 microglial subtype. **C** qPCR analysis of mRNA expressions of CD86 and iNOS in brain tissues across all groups. **D** qPCR analysis of mRNA expressions of CD206 and Arg1 in brain tissues across all groups. **E** Western Blot analysis of TLR4, NF-κB, and MyD88 in the brain post stroke. **F**–**H** Statistical analysis of TLR4 (**F**), MyD88 (**G**), and NF-κB (**H**) protein expressions. Mean ± SEM, n = 5 or 6, one-way ANOVA, vs sham ns: no significance; **P < 0.01, ***P < 0.001; MCAO vs Evobrutinib ns: no significance, ^##^*P* < 0.01, ^###^*P* < 0.001

Next, we hypothesize that Evobrutinib may attenuate the neuroinflammatory response by modulating microglial BTK polarization following its inhibition. Flow cytometry analysis revealed that Evobrutinib decreased the proportion of pro-inflammatory M1 microglia (Fig. 6A) and increased anti-inflammatory M2 microglia (Fig. 6B). Compared with MCAO group, the expression of MHC-II, an antigen presenting marker of microglia, was significantly decreased, while the proportion of CD80^+^ did not change significantly (see Supplementary Fig. 2). Furthermore, qPCR analysis of brain tissue mRNA levels demonstrated that Evobrutinib reduced the expression of M1-associated markers, CD86 and iNOS (Fig. 6C), and increased the expression of M2-associated markers, CD206 and Arg1 (Fig. 6D). These findings suggest that Evobrutinib suppresses BTK activation in microglia, thereby inhibiting M1 and promoting M2 microglial activation. As BTK is involved in the transduction of TLR signaling pathways, and TLR4 is crucial for M1 microglial polarization, we further investigated whether Evobrutinib modulates microglial polarization via the TLR4 pathway. Western Blot analysis of brain tissues from MCAO mice indicated that Evobrutinib significantly downregulated the expression of TLR4, NF-κB, and MyD88 (Fig. 6E).

### Result 5: Evobrutinib inhibits microglial polarization via the TLR4/MyD88/NF-κB signaling pathway

**Fig. 7:**
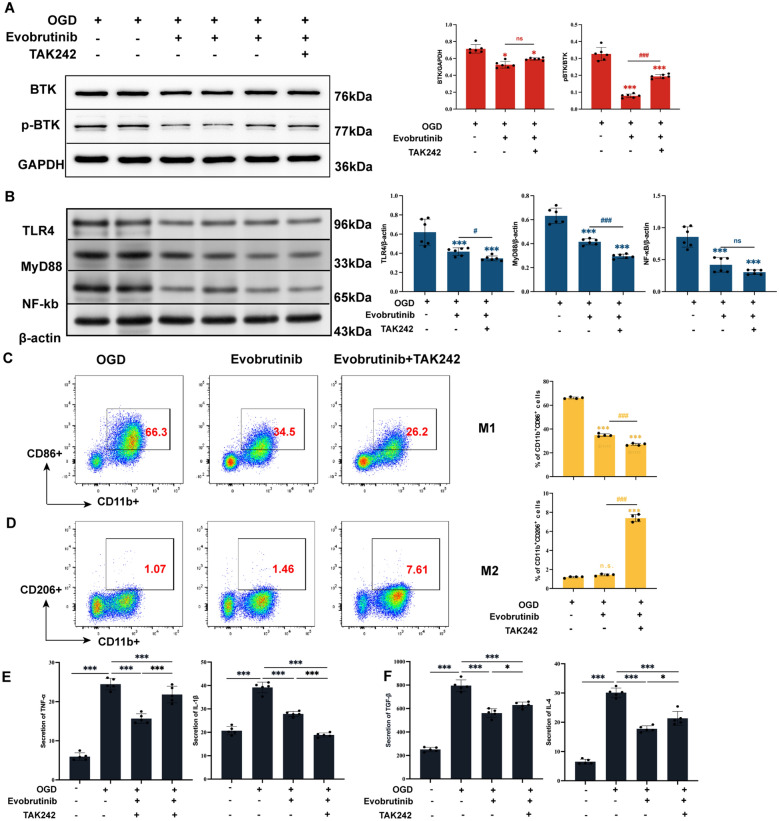
Evobrutinib inhibits M1 microglial polarization via the TLR4/MyD88/NF-κB pathway. **A** Western Blot analysis of BTK and pBTK protein expression levels in microglia from different groups after OGD. **B** Western Blot analysis of TLR4, NF-κB, and MyD88 expression levels in microglia from different groups after OGD. **C** Flow cytometry analysis of the effect of Evobrutinib and TAK242 on M1 microglial polarization. **D** Flow cytometry analysis of the effect of Evobrutinib and TAK242 on M2 microglial polarization. **E**, **F** ELISA detection of pro-inflammatory cytokines TNF-α, IL-1β (**E**) and anti-inflammatory cytokines TGF-β, IL-4 (**F**) levels in the supernatant of microglia after OGD and Evobrutinib treatment. Mean ± SEM, n = 4, 5 or 6, one-way ANOVA, vs sham, ns: no significance, *P < 0.05, ***P < 0.001; MCAO vs Evobrutinib ns: no significance, ^#^*P* < 0.05, ^###^*P* < 0.001

Next, we further investigated the mechanism of Evobrutinib on microglial polarization in vitro. Evobrutinib and TAK-242 were used to inhibit BTK and TLR4 expression in microglial cells, respectively. Western Blot results revealed that the Evobrutinib group exhibited significant inhibition of both BTK and pBTK expression. Co-treatment with Evobrutinib and TAK-242 did not markedly alter BTK protein levels (Fig. 7A). However, this combination more significantly downregulated the expression of TLR4, MyD88, and NF-κB compared to the Evobrutinib-only group (Fig. 7B), suggesting that the TLR4/MyD88/NF-κB pathway might be downstream of BTK. Flow cytometry demonstrated a reduced proportion of M1 microglial polarization in the Evobrutinib group relative to the OGD group, with a further decrease in CD86^+^ microglia and an increase in CD206^+^ microglia observed in the TAK-242 co-treatment group (Fig. 7C, D). ELISA analysis indicated that pro-inflammatory cytokine levels (IL-1β and TNF-α) were elevated in OGD-treated microglia, but significantly reduced under Evobrutinib and combined TAK-242 stimulation (Fig. 7E). Additionally, the expression of anti-inflammatory cytokines (IL-4 and TGF-β) was upregulated (Fig. 7F), with the combined inhibition group showing the lowest secretion levels of pro-inflammatory cytokines. These findings suggest that Evobrutinib may inhibit the M1 microglia-mediated inflammatory response through the TLR4/MyD88/NF-κB pathway.

## Discussion

In this study, we explored the efficacy of Evobrutinib, a third-generation BTK inhibitor, in an animal model for ischemic stroke treatment for the first time. Our results indicated that Evobrutinib can ameliorate neural damage and inflammation in mice with cerebral ischemia. This therapeutic effect appears to be mediated by the suppression of M1 microglial polarization via the TLR4/MyD88/NF-κB signaling pathway (Graphical Abstract).

BTK is a pivotal protein kinase that regulates the growth and development of B cells and their receptor signaling pathways. It is upregulated during inflammatory and immune responses, playing a crucial role (Leitinger and Kaplan 2022). BTK inhibitors are predominantly utilized in treating autoimmune and inflammatory diseases. Ibrutinib, the first FDA-approved BTK inhibitor, has demonstrated efficacy in treating B cell-related malignancies (Rozkiewicz et al. 2023). However, its affinity for Cys481 cysteine residues in kinases such as those from the Tec family, epidermal growth factor receptor, and Janus kinase leads to off-target effects, contributing to adverse events like atrial fibrillation and hypertension (Patel et al. 2017). Subsequent generations of BTK inhibitors, particularly the third generation, have improved safety profiles due to their reversible binding to BTK through hydrogen bonding and hydrophobic interactions at sites other than Cys481 (Tam and Thompson 2024; Ran et al. 2022; Liu et al. 2021). Additionally, the orally administered small molecule Evobrutinib, which can permeate the BBB, has shown therapeutic efficacy in treating multiple sclerosis, a chronic inflammatory demyelinating disease of the central nervous system (Geladaris et al. 2022; Saberi et al. 2023; Montalban et al. 2019). Nonetheless, the capacity of Evobrutinib to alleviate neuroinflammation and confer neuroprotection post-stroke remains less explored. In a study by Maximilian et al., increased BTK expression in brain microglia was observed in a mouse model of stroke (Ito et al. 2015). Our experiments corroborate this finding, with results from TTC staining, behavioral assessments, and pathological evaluations showed that Evobrutinib substantially reduced cerebral infarct size, enhanced motor function recovery, and improved pathological outcomes in ischemic mice. These findings robustly supported the hypothesis that Evobrutinib could be an effective treatment for IS.

In addition to its use in EAE, Evobrutinib has been integrated into treatment strategies for intractable inflammatory diseases such as systemic lupus erythematosus and rheumatoid arthritis (Montalban et al. 2023; Hundelshausen and Siess 2021). Our findings confirmed that Evobrutinib also exerted a significant inhibitory effect on neuroinflammation following IS. Specifically, it reduced neutrophil infiltration and promoted the secretion of anti-inflammatory cytokines in ischemic mice. Previous research on other BTK inhibitors also supports their role in promoting apoptosis of inflammatory cells and inhibiting their activation. At the cellular level, most studies have focused on macrophages or the microglial cell line, BV2. For instance, Ibrutinib has been shown to attenuate LPS-induced pro-inflammatory cytokine secretion in BV2 cells in vitro (Nam et al. 2018). Additionally, in the context of acute lung injury (Li et al. 2022) and pulmonary hypertension (Yu et al. 2022), BTK inhibitors have been found to markedly enhance anti-inflammatory polarization of macrophages. Therefore, in this study, we further explored the effect of Evobrutinib on microglial polarization in the brain post-stroke, a crucial cellular event that influences the inflammatory process in stroke. Our results demonstrated that Evobrutinib treatment significantly downregulated the proportion of M1-subtype microglia and increased the proportion of M2-type microglia in MCAO mice. Changes in the expression of transcription markers for M1 and M2 microglia corroborate these results.

The polarization of microglial cells is a complex process influenced by multiple interacting factors. For instance, inhibition of the mitochondrial electron transport chain can elevate intracellular reactive oxygen species (ROS) concentrations, promoting M1 microglial polarization (Liu et al. 2020b). Wilms tumor suppressor 1-related protein has been shown to enhance the transcription of pro-inflammatory factors in M1 microglia through the modulation of SIRT1 expression (Hsu et al. 2023). Additionally, the Toll-like receptor (TLR) signaling pathway is well known to regulate the pro-inflammatory functions of microglia across various diseases (Heidari et al. 2022; Yang et al. 2020; Reusch et al. 2021). Studies have shown that BTK acts as a regional binding protein of the Toll/IL-1 receptor, which can activate NF-κB—a key downstream inflammatory signal—via TLR4. This process is mediated through the phosphorylation of MyD88, facilitated by BTK (Zheng et al. 2021). Therefore, we hypothesized that the TLR4/MyD88/NF-κB pathway might be the critical pathway through which Evobrutinib inhibits the pro-inflammatory polarization of microglia following stroke. In vitro experiments indicated that Evobrutinib treatment significantly reduced BTK expression and the activation of TLR4 pathways in mouse microglial cells induced by OGD. Furthermore, the addition of the TLR4 pathway inhibitor, TAK242, to Evobrutinib treatment markedly decreased the expression of TLR4, MyD88, and NF-κB, and altered the microglial polarization by reducing the proportion of M1 microglia and increasing M2 microglia compared to treatment with Evobrutinib alone. These findings suggest that Evobrutinib may mitigate neuroinflammation following stroke by inhibiting microglial polarization mediated by the TLR4/MyD88/NF-κB pathway. Current clinical strategies rely on free radical scavengers and oxidative stress inhibitors, which demonstrate therapeutic efficacy primarily during the hyperacute phase of stroke (< 3 h post-onset) (Paul and Candelario-Jalil 2021; Haupt et al. 2023). In contrast, Evobrutinib exhibited notable subacute-phase (24–72 h) anti-inflammatory efficacy. For future clinical implementation, combination therapy integrating Evobrutinib with conventional agents could be considered to synergistically mitigate secondary ischemic injury through mechanistic complementarity. Furthermore, advanced delivery platforms employing nanocarrier-mediated targeted systems may enhance microglial specificity of Evobrutinib, potentially optimizing its blood–brain barrier penetration efficiency and accelerating clinical translation.

Currently, the optimal control targets for different functional subtypes of microglia after stroke, including their numbers and proportions, remain uncertain, and targeted in vivo drug delivery methods to microglia are lacking. Since CNS microglia are the primary cells expressing BTK, Evobrutinib, which targets microglial BTK, may offer neuroprotective effects post-stroke. However, our study had certain limitations. While microglia polarization occurs early, we did not assess long-term outcomes of Evobrutinib treatment on ischemic stroke, including treatment duration and dosing implications. Future work should extend the observation window to comprehensively assess its long-term safety profile. Concurrently, research optimizing dosing strategies may yield novel insights to facilitate the clinical translation of Evobrutinib. Additionally, since BTK is a crucial kinase across multiple receptors signaling pathways, inhibiting its expression in microglia via Evobrutinib might also impact other immune cell functions. This suggests that the effects of Evobrutinib on stroke may extend beyond microglia, potentially influencing overall outcomes and prognoses adversely. Further investigation into Evobrutinib’s effects on other immune cell populations involved in the pathogenesis of IS may elucidate its multifaceted mechanisms of action.

In conclusion, our preliminary studies indicate that Evobrutinib can inhibit M1 microglial polarization via the TLR4/MyD88/NF-κB pathway, mitigate neuroinflammation, and potentially serve as a therapeutic agent for ischemic stroke. As a potential therapeutic target, more comprehensive research on the roles and mechanisms of BTK and BTK inhibitors in stroke is warranted in the future to provide new insights and strategies for clinical stroke treatment.

## Supplementary Information

Supplementary file 1: Fig1 A: Specific gating strategy for flow cytometry of macrophages and B cells in spleen. B, C: Flow cytometry analysis of pBTK expression levels in macrophages (B) and B cells (C) in spleen. Statistical analysis revealed no significant differences in pBTK expression between macrophages and B cells in the spleen. Mean±SEM, n=5, one-way ANOVA, vs sham ns: no significance; MCAO vs Evobrutinib ns: no significance.

Supplementary file 2: Fig 2 A,B: Flow cytometry analysis of MHCII^+^ and CD80^+^ expression in microglia after Evobrutinib treating. Statistical analysis revealed no significant differences in CD80^+^ expression between MCAO and Evobrutinib group while the expression of MHCII^+^ microglia decreased in Evobrutinib group compared to MCAO. Mean±SEM, n=5, one-way ANOVA, vs sham: * P < 0.05; MCAO vs Evobrutinib, ### P< 0.001,ns: no significance.

## Data Availability

The data that support the findings of this study are available on request from the corresponding author, H.L., upon reasonable request. And all additional files are included in the manuscript.
