# Supplementary figures and images for: Evobrutinib mitigates neuroinflammation after ischemic stroke by targeting M1 microglial polarization via the TLR4/Myd88/NF-κB pathway
Source: Mol Med. 2025 Apr 22;31:148. doi: 10.1186/s10020-025-01203-8 (PMC12016189; doi:10.1186/s10020-025-01203-8)

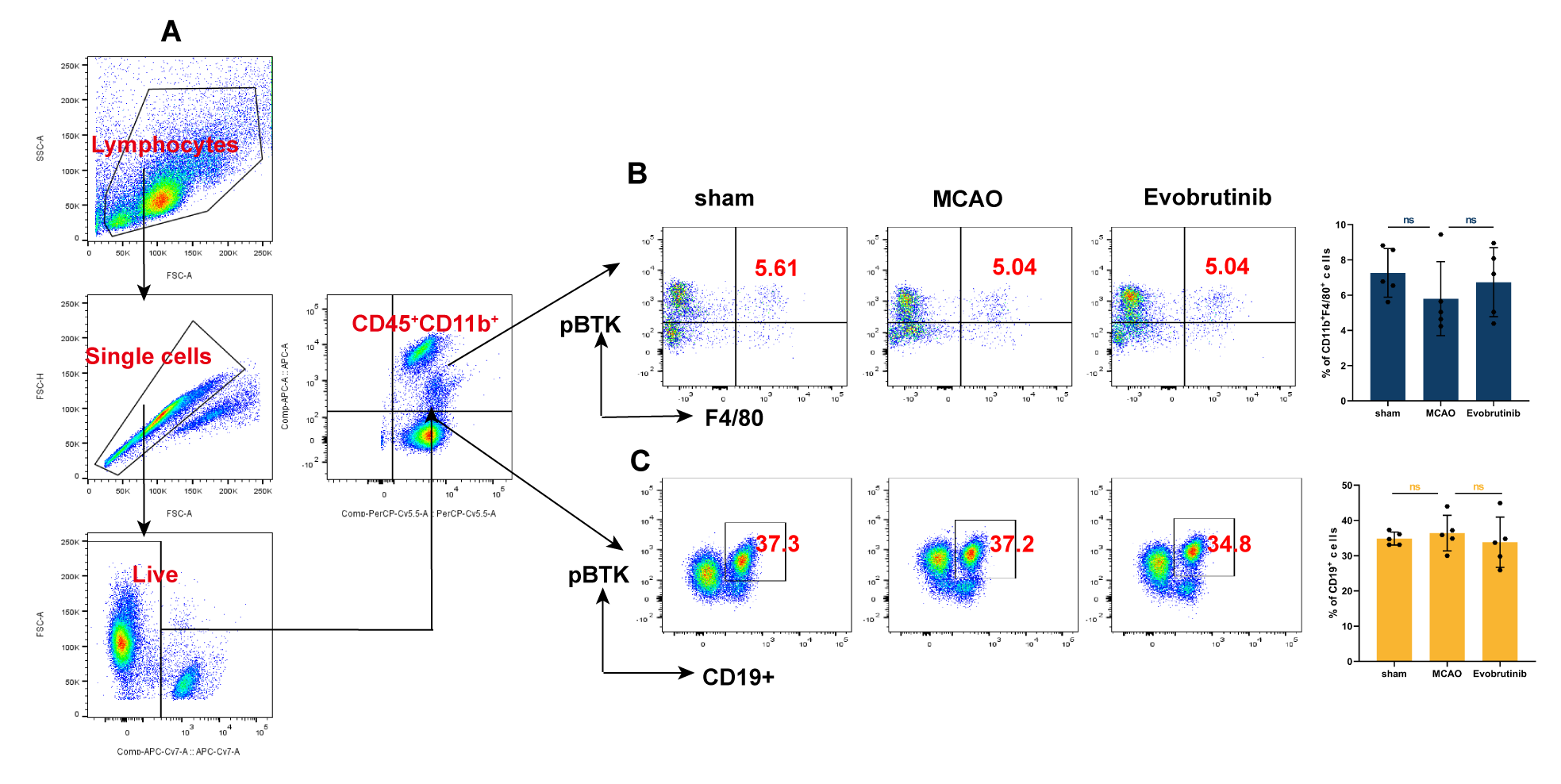

Supplement: Supplementary file 1 — Fig1 A: Specific gating strategy for flow cytometry of macrophages and B cells in spleen. B, C: Flow cytometry analysis of pBTK expression levels in macrophages (B) and B cells (C) in spleen. Statistical analysis revealed no significant differences in pBTK expression between macrophages and B cells in the spleen. Mean±SEM, n=5, one-way ANOVA, vs sham ns: no significance; MCAO vs Evobrutinib ns: no significance. [file 10020_2025_1203_MOESM1_ESM.tif]

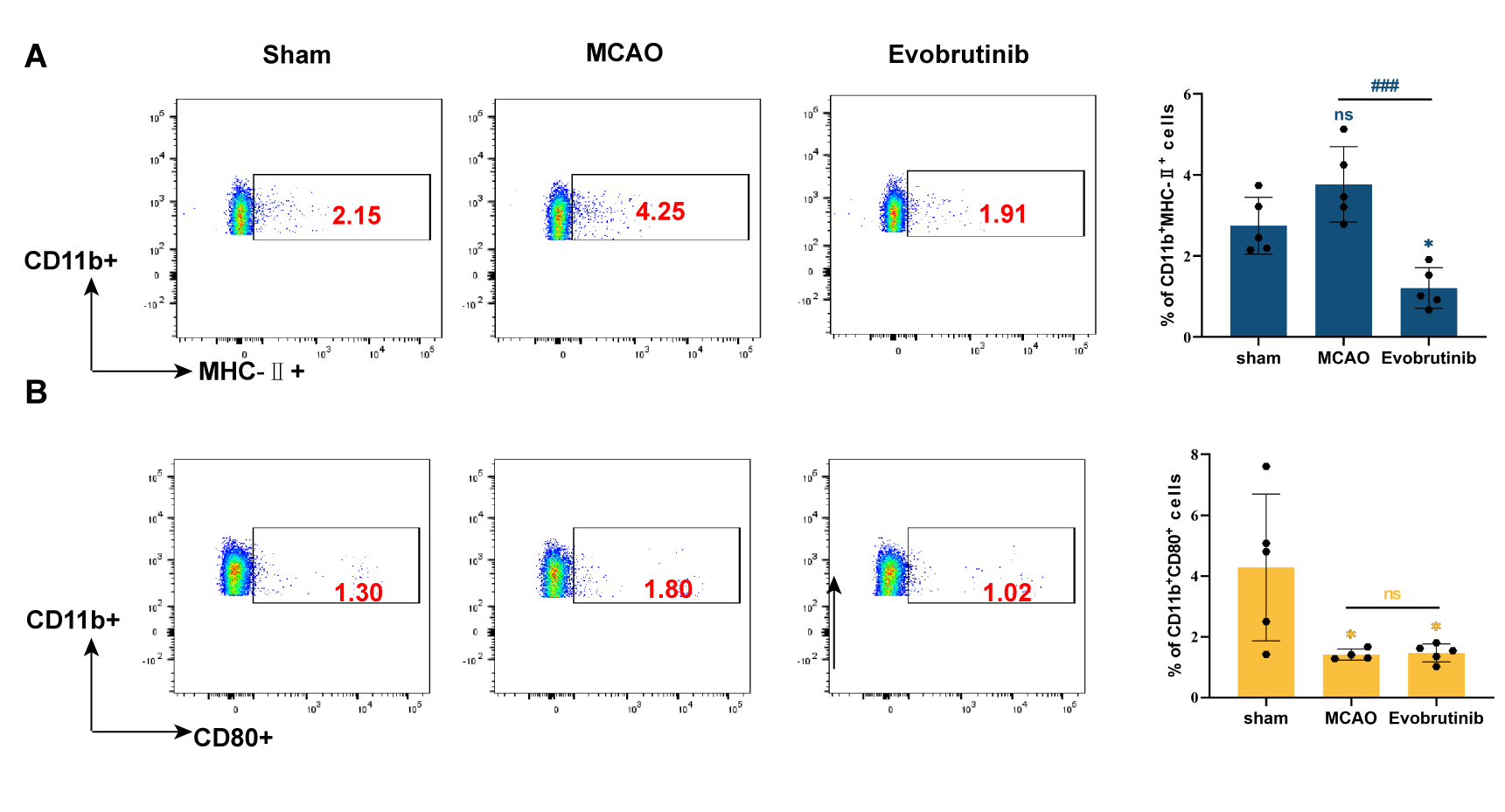

Supplement: Supplementary file 2 — Fig 2 A,B: Flow cytometry analysis of MHCII+ and CD80+ expression in microglia after Evobrutinib treating. Statistical analysis revealed no significant differences in CD80+ expression between MCAO and Evobrutinib group while the expression of MHCII+ microglia decreased in Evobrutinib group compared to MCAO. Mean±SEM, n=5, one-way ANOVA, vs sham: * P < 0.05; MCAO vs Evobrutinib, ### P< 0.001,ns: no significance. [file 10020_2025_1203_MOESM2_ESM.tif]
